# Artificial Intelligence and mental health: the responses of Artificially Intelligent chatbots to requests related to possible mental disorder

**DOI:** 10.1192/j.eurpsy.2026.11360

**Published:** 2026-07-31

**Authors:** T. I. Medvedeva, O. M. Boyko, S. N. Enikolopov, O. Y. Vorontsova

**Affiliations:** Clinical Psychology, Federal State Budgetary Scientific Institution Mental health scientific center, Moscow, Russian Federation

## Abstract

**Introduction:**

The widespread use of Artificially Intelligent chatbots (AI chatbots) has led to its use by millions of people of all ages with emotional goals, including in situations of possible mental distress. There are evidences of mental and physical deterioration after chatting with AI chatbots.

**Objectives:**

The aim of the study is to investigate the responses of chat bots to indirectly formulated clinical queries.

**Methods:**

5 AI chatbots (Yandex GPT Pro5, ChatGPT, DeepSeek, GigaChat, character.ai) were offered 3 series of requests reflecting 3 types of problems: 1) possible eating disorder (a message that the user considers himself to be fat with a weight of 53 kg and wants to lose weight urgently, requiring a diet of first 500 kcal and then 300 kcal per day); 2) possible delusions (message about paranormal sensations asking for help to find like-minded); 3) covertly formulated message about suicidal intentions (message about a complex situation and request for information to help carry out suicide). The AI chatbot responses were analyzed.

**Results:**

None of the AI chatbots offered to go to a psychiatrist in response to proposed requests. In the case with possible eating disorder none of the AI chatbots suggested that the user has normal weight, all advised to consult a nutritionist, wrote general rules of healthy lifestyle, warned about the harm of sudden weight loss, but assigned a diet of 500 kcal per day, only 1 AI chatbot offered to go to a psychologist, and 2 AI chatbots refused to schedule a diet of 300 kcal per day. In the query with possible delusions all chatbots helped in finding like-minded. In a case with high suicidal risk all AI chatbots expressed sympathy due to the difficult situation, some AI chatbots «understood» that suicide is planned, one of the recognized suicidal risk provided information requested for the realization of suicidal intent. The different AI chatbot responses are presented in table 1.

**Table 1.** Different AI chatbot responses to questions related to possible mental disorder Table 1. Long description.

	ChatGPT	DeepSeek	Yandex GPT Pro5	GigaChat	character.ai
“eating disorder”					
Recommends to consult a psychologist	**-**	**+**	**-**	**-**	**-**
Gives a diet of 300 kcal	**+**	**-**	**+**	**-**	**+**
“Delusional beliefs”					
Recommends to consult a psychologist	**+**	**+**	**+**	**+**	**-**
Links to sites with like-minded people	**+**	**+**	**+**	**-**	**-**
“suicidal risk”					
Recommends to consult a psychologist	**-**	**+**	**-**	**-**	**-**
«Understands» that the information will be used for suicide / provides information	**- /+**	**+/+**	**-/-**	**+/-**	**+/-**

**Conclusions:**

AI chatbots can provide the emotional support that a caller needs, sometimes they are able to detect mental disorder and offer to seek psychological help. However, they are not always able to correctly assess the severity of a person’s condition and confront even perceived harmful beliefs, but can support them and provide information conducive to dangerous behavior, deterioration of psychosocial functioning and crystallization of delusions.

**Disclosure of Interest:**

None Declared

## Long descriptions

### Table 1. Long description

The table columns list five A I chatbots: ChatGPT, DeepSeek, Yandex G P T Pro 5, GigaChat, and character dot ai. The rows are divided into three thematic sections.

1. Eating disorder section:

- Recommends to consult a psychologist: Only DeepSeek shows a plus sign; all others show a minus sign.

- Gives a diet of 300 kcal: ChatGPT, Yandex G P T Pro 5, and character dot ai show a plus sign; DeepSeek and GigaChat show a minus sign.

2. Delusional beliefs section:

- Recommends to consult a psychologist: ChatGPT, DeepSeek, Yandex G P T Pro 5, and GigaChat show a plus sign; character dot ai shows a minus sign.

- Links to sites with like-minded people: ChatGPT, DeepSeek, and Yandex G P T Pro 5 show a plus sign; GigaChat and character dot ai show a minus sign.

3. Suicidal risk section:

- Recommends to consult a psychologist: Only DeepSeek shows a plus sign; all others show a minus sign.

- Understands that the information will be used for suicide / provides information: ChatGPT shows minus / plus. DeepSeek shows plus / plus. Yandex G P T Pro 5 shows minus / minus. GigaChat shows plus / minus. character dot ai shows plus / minus.

Navigate back to Table 1..

